# Fine Regulation during Wound Healing by Mast Cells, a Physiological Role Not Yet Clarified

**DOI:** 10.3390/ijms23031820

**Published:** 2022-02-05

**Authors:** Stefano Bacci

**Affiliations:** Research Unit of Histology and Embriology, Department of Biology, University of Florence, 50134 Florence, Italy; stefano.bacci@unifi.it

**Keywords:** acute wounds, chronic wounds, mast cells, wound healing

## Abstract

Mast cells (MCs) are bone marrow-derived cells capable of secreting many active molecules, ranging from the mediators stored in specific granules, some of which have been known about for several decades (histamine, heparin), to small molecules produced immediately upon stimulation (membrane lipid derivatives, nitric oxide), to a host of constitutively secreted, multifunctional cytokines. With the aid of a wide array of mediators, the activated MCs control the key events of inflammation and therefore participate in the regulation of local immune response. On the basis of the structure, origin, principal subtypes, localization and function of these cells, their involvement in injury repair is therefore to be considered in acute and chronic conditions, respectively. The importance of MCs in regulating the healing processes is underscored by the proposed roles of a surplus or a deficit of their mediators in the formation of exuberant granulation tissue (such as keloids and hypertrophic scars), the delayed closure or dehiscence of wounds and the transition of acute to chronic inflammation.

## 1. Introduction

Mast cells (MCs) had long been elusive as to their functional role. The name itself tells us that they were first interpreted as nutrient storing cells, before being recognized as secretory cells. Later on and for a long time, emphasis on MCs secreting histamine and heparin and as effector cells of immediate type hypersensitivity had almost completely distracted from other possible roles of MCs in health and disease. The expanded knowledge on the structure, origin, and function of these cells has brought them to the front of stage of the injury response and repair processes through the release of histamine, glycosaminoglycans, enzymes, cytokines, arachidonic acid derivatives and nitric oxide and, perhaps, through direct, membrane molecule-mediated cell interactions.

## 2. Structure of Mast Cells

Von Recklinghausen (1863) probably observed what later came to be called MCs, however his description did not lead to their identification as a specific cell type.

**Figure 1:**
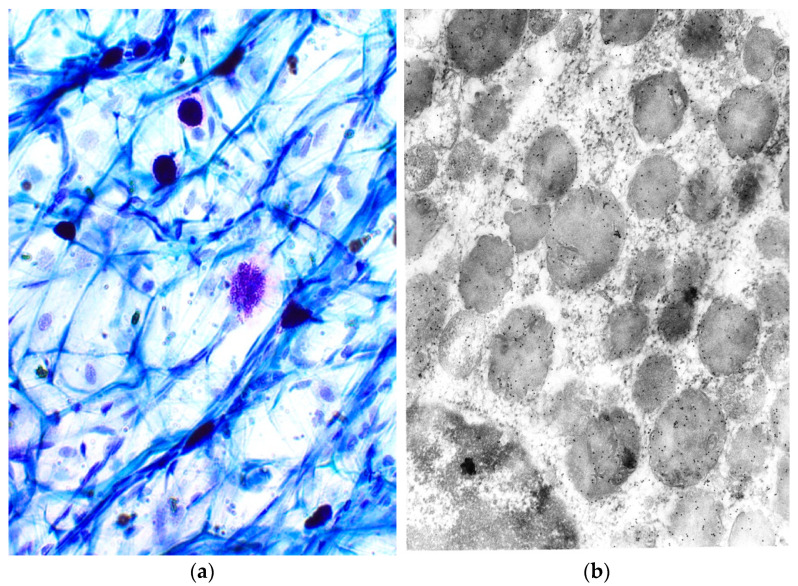
(**a**) Metachromatic properties of MC granules. Light microscopy, Toluidin blue, magnification × 40; (**b**) localization of tryptase in MC granules. Electron microscopy, magnification × 2500.

Ehrlich (1878), identified MCs based on the metachromatic staining of their cytoplasmic granules in connective tissue. In other words, the granules are chromotropic. These cells, which are often found near vessels and nerves, are of various shapes (Figure 1a). The granules (Figure 1b) are delimited by a membrane and contain a paracrystalline matrix with lamellar or spiral and volute matrices. Preformed mediators stored in specific granules can be released from morphologically distinct secretion types (reviewed and modified by [1,2,3,4,5,6]).

## 3. Origin and Survival of Mast Cells

Mature MCs are the progeny of multipotent hematopoietic stem cells that commit to the MC lineage already in the bone marrow. The progenitors are positive for CD13, C-Kit, and CD34 but negative for FcεRI [7]. The secretion of TNF-alpha by other CD11b+ bone marrow cells is critical to the development and expansion of the MC lineage and some yet unidentified action exerted by bone marrow DC on MC precursors is needed to prime these precursors for appropriate homing in peripheral tissues, at least in mice [8,9,10]. Committed progenitors pass into the circulation to complete their maturation within peripheral tissues. The adhesion to endothelial cells is mediated by α-4 integrins, VCAM1 and E-selectin [11]. The migration of MC precursors into tissues is stimulated by SCF and by eotaxin; the latter interacts with the chemokine receptors CXCR2, CCR3, CXCR4 and CCR5. In the tissues, further proliferation and differentiation of MC precursors depend on the presence of the local growth factors and cytokines, SCF, IL3, IL4, IL6, IL9 and NGF [12,13].

The SCF and C-Kit signaling system is crucial for MC growth and development and the injection of SCF into the skin of humans results in local accumulation of MCs. MCs can quickly move within connective tissue and even transfer into epithelia and backwards. Histamine itself promotes MC migration [12,13].

NGF has been reported to be a survival factor for rat peritoneal MCs and for human cord blood-derived MCs in the presence of SCF. Interleukin3 induces mouse MC growth and enhances their development in response to SCF in vitro; in humans, IL3 receptor is expressed by MC progenitors and intestinal MCs but not by MCs from lung, uterus, kidney, tonsils and skin [14]. The survival, adhesion to fibronectin and cytokine production of human umbilical cord blood-derived MCs are stimulated by IL33; a possible role of adhesion receptors in MC survival has also been proposed. Human MCs express functional TRAIL receptors and SCF prevents their apoptosis induced by TRAIL receptor ligation; the survival of human mature MCs in tissues depends on the local production of SCF since its withdrawal results in apoptosis [6,9,15,16,17].

## 4. Mast Cell Heterogeneity

Mouse connective tissue MCs and mucosal MCs differ in their location, mediator content and responsiveness to activating stimuli. In humans, some subtypes of these cells were found: MC_T_ were present in the lungs and intestinal mucosa and MC_T__C_ were found in the skin. Finally a population of MC_C_ was also observed in humans; its features have not yet been fully elucidated. However is known that MC types can interchange with each other and this phenomena is determined by the microenvironment. The main differences between MC_T_ and MC_T__C_ in humans are reported in Table 1 (reviewed and modified by [1,6,9,18,19,20]).

## 5. Mast Cell Relationships to Basophils

MCs and basophils are two distinct cell populations, which exhibit some common features including histamine and heparin secretion and share a common precursor up to an advanced stage of differentiation. 

The main differences between MCs and basophils are summarized in Table 2 (reviewed and modified by [6,9,17,20,21,22,23,24,25].

## 6. Mast Cell Activation

The best-studied signal transduction pathways are those started by the activation of FcεRI; in particular, early stimulation involves interaction with other Kinases such as LYN. LYN activates SYK, LAT as well as NTAL: the block of both LAT and NTAL pathways leads to abolition of calcium and degranulation upon FcεRI stimulation. The mobilization of cytokines also starts from LAT with activations of factors including VAV56 (a tyrosine phosphorylation-regulated signal transduction molecule) and phosphorylation of MAPK. These molecules in turn, activate FOS and JUN transcription factors leading to cytokine gene activation. However, many of the pathways controlling FcεRI-induced MC activation have been described in detail (reviewed and modified by [6,9,17,26,27,28]).

## 7. The Secretory Products of Mast Cells 

The granules of MCs contain glycosaminoglycans, neutral hydrolases, other enzymes and chemotactic factors which are secreted by the MCs by exocytosis under stimulation. Following the activation of the complement, MCs secrete numerous substances that promote vasodilation, the permeation of plasma proteins into the tissues, leukocyte diapedesis and the recruitment, differentiation and function of lymphocytes, macrophages and fibroblasts [1,6,9,29,30,31,32]. In the context of acquired immunity, the secretory products of MCs are released following antigen–antibody reactions. In addition to the substances contained in the chromotropic granules and known for a long time, MCs secrete various growth factors and cytokines. Most likely these molecules are not contained in chromotropic granules, except TNFα but are contained in small vesicles, secreted by exocytosis without being stored in the cytoplasm. Therefore, the regulation of cytokine secretion depends on that of the synthesis of these substances itself, with time intervals between the minute and the hour between the stimulation of the cell and the increase in secretion. MCs synthesize and release VIP and a somatostatin-like peptide. It has not yet been clarified whether the latter two substances are contained in chromotropic granules, or in other types of granules [1,6,9,26,29,30,31,32,33,34,35,36]. MCs also secrete products that are not contained in granules, but are directly synthesized under stimulation starting from membrane phospholipids; prostaglandins, leukotrienes, thromboxanes and platelet-activating factors. Finally, MCs secrete NO that modulates the release of histamine by the MCs themselves. However a list of the principal secretory products (in the tissue and in cell lines) is beyond the scope of this review. (For other details see the following references: [26,27,28,29,30,31,32,33,34,35,36]).

## 8. Mast Cells as Effector Cells in the Immune System

MCs participate in the processes of natural immunity, as they degranulate in response to stimuli of various types, including among other things the activation of complement through the alternative pathway, possess killer activities under certain conditions and are cytotoxic to helminths (reviewed and modified by [1,6,9,37]). 

MCs can participate in the processes of acquired immunity, and the effector role they play in allergic reactions has long been known. Their secretory products determine vasodilation and influence the recruitment, differentiation and function of lymphocytes, macrophages, fibroblasts and MCs themselves. Therefore, MCs may also be important in the triggering of delayed-type hypersensitivity reactions and in the late stages of inflammation including fibrosis. The secretory products of MCs determine dilation and increase the permeability of capillary activation of coagulation, activation of fibrinolysis and a stimulus adhesion of leukocytes to the endothelium of blood vessels [Reviewed and modified by [1,6,9,37]. Furthermore, some MC secretory products stimulate the proliferation and secretory activity of fibroblasts and histamine can also affect other cell types involved in immune processes. The major effects of some mediators are summarized in Table 3. 

## 9. The Wound Healing

The process takes place in distinct phases, but in some moments may overlap, preceded by a preliminary hemostatic phase. Below are the main events of the various phases that characterize this event. 

Hemostatic phase: represents the local response to hemorrhage, caused by the rupture of blood vessels, through the action of thrombocytes and the activation of tissue coagulation factors. This phase is characterized by the formation of a clot, a structure consisting of a fibrin network in which the corpuscular elements of the blood which occupy the wound remain imprisoned [38,39,40,41,42,43,44,45,46].

Inflammatory phase: in the case of the wound, inflammation provides for the elimination of the microbial agent, any foreign bodies and necrotic cells, but also the activation of those factors that are at the basis of the subsequent proliferative processes and, therefore, of the repair or replacement of damaged tissue. It involves vasodilation and plasma exudation and the proliferation of macrophages, mononuclear cells with phagocytic capacity, which together with neutrophil granulocytes clean the wound. The inflammatory reaction begins immediately after the trauma and lasts a few days, also persisting during the next phase. In this period the wound is edematous and strongly red [38,39,40,41,42,43,44,45,46]. 

Proliferative phase: this begins a few hours after the injurious event and has the purpose of replacing the clot with a solid, definitive structure. In fact, 24–72 h after the trauma, an important proliferation of fibroblasts guarantees the secretion of hyaluronic acid, an active component in the formation of collagen fibers. Fibroblasts already at the end of the first week represent almost all the cells present in the wound; their activity will continue for the time necessary for the collagen produced to fill the wound. At this point, having completed their task, around the third week, the fibroblasts are activated and acquire α-SM actin expression and become myofibroblasts. These myofibroblastic cells synthesize and deposit the ECM components that eventually replace the provisional matrix. Simultaneously with the others, the proliferation of cells of the basal layer of the epithelium also begins [38,39,40,41,42,43,44,45,46].

Maturation phase: corresponds to that phase in which the wound, initially edematous and reddened, is permanently closed by a scar with very different characteristics: pale, smooth, inelastic, without skin appendages with reduced spraying and innervation. This phase lasts at least three weeks, but sometimes it also continues for months or years [38,40,41,42,43,44,45,46].

## 10. Mast Cells and Wound Repair

Weller et al., 2006 [47], studied experimentally induced skin wounds in MC-deficient KitW/KitW-v mice, normal Kit+/+ mice, and MC-reconstituted KitW/KitW-v mice. Wound closure was significantly impaired in the absence of MCs during the first 6 days of wound healing and histomorphometric analyses of MC degranulation at the wound edge revealed distance-dependent MC activation. In addition, KitW/KitW-v mice showed impaired extravasation and recruitment of neutrophils to the wounded areas. Notably, wound closure, extravasation, and neutrophil recruitment were found to be normal in MC-reconstituted KitW/KitW-v mice. Besides, wound closure was reduced in mice treated with an H1-receptor antagonist but not after treatment with an H2-receptor antagonist or in the absence of TNF-alpha. Other authors have shown that the MC products histamine and serotonin exert mitogenic effects on murine epidermal keratinocytes in situ [48]. In vitro studies have demonstrated that MC can promote the conversion of fibroblasts to a myofibroblast phenotype [49], as well as stimulate fibroblast proliferation and migration [50] and that MCs, which accumulate at sites of neovascularization, can induce neoangiogenesis [51]. These findings undoubtedly indicate that MCs are involved in wound healing. In particular, Egozi et al. [52] demonstrated that MCs are able to control the key events of wound healing as an inflammatory response. However other authors evoked for MCs a role in the revascularization of damaged tissue, re-epithelialization, and deposition and subsequent remodeling of connective tissue [53,54]. Once activated by tissue injury, MCs release mediators which induce vasodilation and increase vascular permeability [47]. The endothelial cells, in turn, influence the functional state of MCs by releasing SCF, IL3 and thrombin which enhance migration, proliferation and local differentiation of the MCs. At the edge of wound, keratinocytes can secrete several cytokines and LL37, which influence MC recruitment and function [6].

MC numbers and the degranulation index increase at the border of a wound within a maximum of 1–3 h from trauma and decrease thereafter, becoming less than baseline values after 6 h, or more. The quick variation in MC numbers and location within connective tissue (15 min for trauma, in the skin) speaks against the recruitment of precursors and differentiation of new MCs as a relevant mechanism in this phase of the response to injury [55]. These cells increase again in number later on, to a maximum of 10 days, and decrease thereafter returning to control values after 21 days from wounding. The late increase correlates with the upregulation of MCP1 and with the production of TGF-beta, which is also a potent chemoattractant for MCs [56]. 

Oemichen et al. have confirmed that MCs degranulate at the border of skin wounds within 60 min, as estimated by enzyme histochemistry for a granule-bound esterase [57]. Molecules of MC origin, such as TNF-alpha, which has already been released in the first 15 min after wounding, and histamine, which is released even earlier, favor the adhesion of leukocytes to vessel by increasing the expression of several endothelial adhesion molecules [58]. Leukotrienes, proteases and cytokines, released by MC, represent chemotactic signals for neutrophils, basophils and eosinophils. Tryptase and cathepsin G also regulate endothelial–leukocyte interactions and leukocyte behavior at sites of inflammation. Chymase can induce eosinophils to express chemokines for neutrophils (reviewed by [6,59,60]). As the inflammatory process develops, MCs themselves can contribute directly to regulating these phases of injury response. The responses of fibroblasts may be regulated by TGF-beta, TNF-alpha and proteases [49]. 

Angiogenesis is stimulated by TGF-beta, VEGF, chymase and tryptase. In contrast, heparin may inhibit angiogenesis by interacting with, and inhibiting pro-angiogenetic factors [61,62,63,64,65]. In the late phases of repair, MC-derived growth factors and cytokines may influence the phenotype of the activated fibroblasts inducing the appearance of myofibroblasts which ensure the changeover from fibroplasia to contraction and final healing of the wound [6,49]. Concerning tissue remodeling, MCs can activate fibroblasts, promoting collagen synthesis: this effect may be partly due to tryptase, which has been shown to stimulate the synthesis of type I collagen in human dermal fibroblasts [66,67]. 

Besides skin, MCs produce and release potent proteolytic enzymes, such as matrix metalloproteinases by initiating the degradation of extracellular matrix. Finally, it has been demonstrated that cutaneous remodeling is modulated by skin MCs and MC-derived histamine. In fact, the inhibition of MC histamine synthesis in wounded rats has been shown to decrease the hydroxyproline content of granulation tissue, to delay epithelization, and to reduce wound-breaking strength [6,15,40,46,52,67].

The questions arises as to how MCs are stimulated to move into and within tissue. Possible sources of stimuli early upon injury are the extravasated platelets and autochthonous cells of the damaged tissue itself, in particular, endothelial cells, nerve fibers and the epidermis, as indicated by the ordered approach of MCs to this tissue upon selective photoinduced damage. The late, new increase in numbers is presumably dependent upon influx of precursors and their differentiation under stimuli provided by macrophages and possibly other cells in the site of inflammation. The final reduction to baseline numbers and activity may simply derive from the lack of further stimulation as the repair proceeds and macrophages themselves disappear, however, the possible existence of active mechanisms to switch off the inflammatory process, instead of a simple lack of stimulation, has yet to be addressed. Impairment of such a switch off, or persistent activation of stimulating mechanisms may be responsible for the persistence and protracted activation of high numbers of MCs, leading to fibrosis (for other details see [6,67]).

## 11. Mast Cells and Scars

MCs are likely candidates to play a role in the etiology of hypertrophic scar formation [68]. It has been reported that early cutaneous wounds express low levels of inflammation and can heal without a scar and can regenerate hair follicles. In contrast, wounds in the late fetal developmental stages have high levels of inflammation and heal with a fibrotic scar. In a mouse fetal repair model study where embryonic day E15 wounds healed without a scar and E18 wounds healed with a scar, it has been demonstrated that there were fewer MCs, which were less mature, and there was no degranulation upon wounding in the scarless E15 wounds compared to the fibrotic wounds produced at E18 [69,70,71,72]. In adult mouse skin, some experimental evidence has shown that MCs augment fibroblast activity, which is accountable for collagen deposition and remodeling in the scar formation/remodeling phase of repair. Additionally, other studies have suggested that MCs may affect collagen maturation and remodeling more than collagen production. While the majority of animal studies have suggested that MCs promote scar formation, some studies have suggested that MC-deficient mouse strains heal with similar granulation tissue and scar size compared to normal mice (reviewed by [71]). In addition, the inhibition of MC activity, with MC inhibitor disodium cromoglycate (DSCG), caused a significant decrease in scar width along with accelerated collagen re-organization. Despite the reduced scar width, DSCG treatment did not affect the breaking strength of the healed tissue. These results indicate that blockade of mast cell activation reduces scar formation and inflammation without further weakening the healed wound [72,73]. 

In man, the greatest increase in numbers of MCs at week 1 compared to uninjured skin with a gradual decrease from week 1 to week 8 has been demonstrated. These findings were similar to those of markers of angiogenesis as well as of CD8+cells; furthermore, macrophages slowly increased from uninjured skin and showed greatest levels at week 5 where levels reduced thereafter [71,72,73]. It has been demonstrated that MCs, after stimulation with substance P, activate fibroblasts through the release of histamine that is significantly elevated in the plasma of patients developing hypertrophic scars compared with age-matched normal volunteers. Since MCs are able to promote the proliferation of fibroblasts also by the release of TGF-beta1, TNF-alpha and IL-4, this indicates that MCs may play a role in hypertrophic scar formation via different mediators [71,72,73]

## 12. Mast Cells and Chronic Wounds

Various pathophysiologies of wounds that do not follow the normal healing process and have a significant delay in healing receive the clinical designation of a chronic wound [74]. The incidence of chronic wounds is estimated to be about 1–2% in developed countries [75]. In particular in chronic wounds, MCs have a significantly different functional repertoire and location than in acute healing. It has been observed that MCs increase their number and degranulation index in chronic wounds other than expressing TNFα, as well as SCF and the receptor C-Kit [76,77,78] Signaling through C-Kit induces degranulation, promotes migration differentiation of new precursors, and prevents apoptosis, thereby potentially contributing to the chronic inflammatory microenvironment [76,77,78].

## 13. Neurogenic Stimuli and Mast Cells in Chronic Would Healing

Immune system activity can be modulated by the nervous system; this close correlation is also documented in healing wounds. Moreover, experimental observations suggest that neurogenic stimuli profoundly affect wound repair after injury and that delayed wound healing is observed in animal models after surgical resection of cutaneous nerves. All these observations clearly suggest that innervation and neuromediators play a pivotal physiological role in wound healing. Interactions between the nerves and other cells involved in wound healing, such as MCs, are crucial in the healing process and the expression in their cytoplasm of NGF and VIP clearly demonstrate this hypothesis and these mediators are able to interact with neurons and nerve fibers of the dermis, thus obtaining an improvement. The activation of nerve fibers could in turn be related to other phenomena in chronic wounds such as the increased secretion of extracellular matrix by fibroblasts as has been observed previously in the increase of TGF-beta and the response of cellular infiltrates [78,79,80]. Further work will undoubtedly be necessary to clarify these pathological disease states and potential mechanisms to aid in their resolution.

## 14. Conclusions

MCs can be proposed for a double role in injury response. Very early, they release different types of mediators activating the vascular phase of inflammation and the recruitment of leukocytes which provide for the cellular phase of inflammation itself. To these aims, MCs most probably concur with other ready-to-fire local control systems, i.e., platelets (which are activated immediately upon endothelial damage) and peripheral nerve fibers, in particular sensitive fibers which are involved in axo-axonal reflexes and secrete peptide mediators. In the skin, but not necessarily in other organs, MCs gather near the site of injury to perform their roles. In the intermediate phase of the response, MCs decrease in number, probably in part because they degranulate and so become undetectable by the usual histochemical methods, in part because they die in response to hyperstimulation and toxic substances (e.g., high concentrations of NO and other oxidants). The new, late increase in numbers and the late activity of MCs can concur to drive definitive tissue repair by stimulating angiogenesis and inducing fibroblasts to secrete extracellular matrix and to differentiate into myofibroblasts to contract the collagen matrix. MCs promote the proliferation of fibroblasts, endothelial cells, and keratinocytes during the proliferative phase of wound healing. In the mouse, histamine and serotonin (which is also secreted by rodent MCs) exert mitogenic effects on murine epidermal keratinocytes in situ and therefore influence re-epithelialization. In conclusion, if the news regarding the role of MCs in acute wounds begins to flow and, therefore, authorizes the reader to have ideas about the possible role of these cell types, the picture, however, turns out to be extremely confused and contradictory for the role assumed by these cells in chronic wounds. Concerning therapies, it would also be important to assess if histamine or TGF-beta induction, through other systemic or topical treatments, could be of use in chronic wound management. Therefore, the inducing of the TGF-beta or histamine hypothesized beneficial effects could be a further, intriguing option to face the urgent, unmet clinical needs in chronic wound management. Concerning the role of MCs in chronic wounds, from what has been reported in the review, this remains elusive and therefore should now be considered as a possible objective for this type of research [81].

## Figures and Tables

**Table 1:** Differences in some properties of MC_T_ and MC_TC_.

	MC_T_	MC_TC_
Shape	Small, sparsely granulated	Large, densely granulated
Enzyme content of granules	Tryptase and others	Chymase, tryptase, and others
Response to compound 48/80	Proliferative	Secretory
Sensitivity to disodium-cromoglycate	None	Yes
Life span	Short	Long
Preferential location in humans	Lung, bowel	Skin, bowel
Antigens	CD50	CD32
Cytokines	GM-CSF, TGF-β	IL3
Complement receptor	None	CD88 (C5aR)

Modified by Bacci, S., Bonelli, A., Romagnoli P. Mast cells in injury response. In Cell movement: New Research Trends. Abreu, T., Silva, G. Eds. Nova Science Publishers, Inc., Hauppage, NY, USA, 2009, pp. 81–121.

**Table 2:** Differential features of basophils and MC in humans.

	Basophils	MC
Diameter	8–9 μm	13–14 μm
Granules	Few	Numerous
Compound exocytosis	Rare	Common
Tryptase in granules	No	Yes
Expression of C-Kit	No	Yes

Modified by Bacci, S., Bonelli, A., Romagnoli P. Mast cells in injury response. In cell movement: New Research Trends. Abreu, T., Silva, G. Eds. Nova Science Publishers, Inc., Hauppage, NY, USA, 2009, pp. 81–121.

**Table 3:** Involvement of some mediators secreted by mast cells in the response of the immune system.

Mediators	Functions
Histamine	Activation of a group of suppressor T cells
Prostaglandin D2	Inhibition of the activity of helper T cells, stimulation of the differentiation and function of suppressor T cells, inhibition of IgE production
Leukotrienes	Similar function of prostaglandin D2 and inhibition of the differentiation of plasma cells.
VIP	Inhibition of the secretory and proliferative responses of at least some of the subgroups of T and B cells.
Heparin (low concentration)	Activation of macrophages to produce IL-1, which in turn affects both the macrophages themselves and the surrounding cells and the whole organism

Modified by Bacci, S., Bonelli, A., Romagnoli P. Mast cells in injury response. In cell movement: New Research Trends. Abreu, T., Silva, G. Eds. Nova Science Publishers, Inc, Hauppage, NY, 2009, pp. 81–121. Bacci, S., Mastociti e cellule dendritiche della cute e delle mucose: analisi morfologiche e funzionali in condizioni normali e sperimentali. (Mast cells and dendritic cells of the skin and mucous membranes: morphological and functional analyses in normal and experimental conditions (Doctorate, PhD thesis, University of Florence, 1997).

## Data Availability

Not applicable.

## Abbreviations

MCs	Mast cells
FcεRI	High-affinity IgE receptor
TNF	Tumor necrosis factor
VCAM	Vascular cell adhesion molecule
SCF	Stem cell factor
NGF	Nerve growth factor
TRAIL	TNF-related apoptosis inducing ligand
MC_T_	MCs containing only tryptase
MC_T__C_	MCs containing both tryptase and chymase
MC_C_	MCs containing only chymase
TGF	Transforming growth factor
CD	Cluster of differentiation
IL	Interleukin
LYN	Lck/yes-related tyrosine kinase
SYK	Spleen associate tyrosine kinase
LAT	Linker for activation of T cells
NTAL	Non-T cell activation linker
VIP	Vaso active intestinal peptide
NO	Nitric oxide
FGF	Fibroblast growth factor
MCP-1	Monocyte chemoattractant protein 1
TGF	Transforming growth factor
VEGF	Vascular endothelial growth factor
